# Forced Running Endurance Is Influenced by Gene(s) on Mouse Chromosome 10

**DOI:** 10.3389/fphys.2017.00009

**Published:** 2017-01-23

**Authors:** Mindaugas Kvedaras, Petras Minderis, Andrej Fokin, Aivaras Ratkevicius, Tomas Venckunas, Arimantas Lionikas

**Affiliations:** ^1^Institute of Sport Science and Innovations, Lithuanian Sports UniversityKaunas, Lithuania; ^2^School of Medicine, Medical Sciences and Nutrition, College of Life Sciences and Medicine, University of AberdeenAberdeen, UK

**Keywords:** electric stimulation, specific force, chromosome substitution strains, exercise, skeletal muscle

## Abstract

Phenotypic diversity between laboratory mouse strains provides a model for studying the underlying genetic mechanisms. The A/J strain performs poorly in various endurance exercise models. The aim of the study was to test if endurance capacity and contractility of the fast- and slow-twitch muscles are affected by the genes on mouse chromosome 10. The C57BL/6J (B6) strain and C57BL/6J-Chr 10^A/J^/NaJ (B6.A10) consomic strain which carries the A/J chromosome 10 on a B6 strain background were compared. The B6.A10 mice compared to B6 were larger in body weight (*p* < 0.02): 27.2 ± 1.9 vs. 23.8 ± 2.7 and 23.4 ± 1.9 vs. 22.9 ± 2.3 g, for males and females, respectively, and in male soleus weight (*p* < 0.02): 9.7 ± 0.4 vs. 8.6 ± 0.9 mg. In the forced running test the B6.A10 mice completed only 64% of the B6 covered distance (*p* < 0.0001). However, there was no difference in voluntary wheel running (*p* = 0.6) or in fatigability of isolated soleus (*p* = 0.24) or extensor digitorum longus (EDL, *p* = 0.7) muscles. We conclude that chromosome 10 of the A/J strain contributes to reduced endurance performance. We also discuss physiological mechanisms and methodological aspects relevant to interpretation of these findings.

## Introduction

Endurance as a component of physical fitness is an important determinant of health and well-being. For instance, the risk of mortality due to cardiovascular disease in obese men is significantly reduced with increasing level of fitness (Lee et al., 1999). Genetic factors account for a substantial portion of endurance capacity with heritability estimates around 50% (Bouchard et al., 1998). Hence, understanding of the underlying genetics might reveal new biomarkers and targets for pharmaceutical interventions to improve fitness and health.

The ability to generate energy via aerobic pathways is a significant determinant of endurance performance. It has been demonstrated that endurance improvement is concomitant with an increase in VO_2_max and activity of the mitochondrial enzymes (Vollaard et al., 2009). Indeed, genes involved in aerobic oxidation and mitochondrial biogenesis may have a substantial influence on endurance capacity. For example, overexpression of PPARdelta (Wang et al., 2004), which stimulates mitochondrial biogenesis in skeletal muscle, or PEPCK-C (Hakimi et al., 2007), which can contribute to the flux through the tricarboxylic acid (TCA) cycle (Burgess et al., 2004), significantly improved running endurance in mice. However, it remains unclear whether these genes play a role in variability in endurance observed in human populations or animal models.

Analyses of the laboratory mouse strains revealed that genetic variability can substantially influence endurance capacity. This influence is evident even when different types of motor activity are explored. For instance, the A/J strain performs poorly in forced running (Lightfoot et al., 2001; Hoit et al., 2002; Courtney and Massett, 2012) and swimming (Kilikevicius et al., 2013) endurance tests compared to other strains including the C57BL/6J (B6). Thus, these two strains provide an interesting model for studying the genetic mechanisms influencing endurance performance.

Chromosome substitution strains, also known as consomic strains, provide a simple model for exploring genetic influences. They consist of a panel of strains where one chromosome of a host strain is replaced by a homologous chromosome of a donor strain (Matin et al., 1999; Singer et al., 2004; Ishii et al., 2011). Phenotypic differences between host and a consomic strain implicate the variants of genes residing on a particular chromosome in their etiology. A genome wide screening of a panel of consomic strains would still require a substantial number of animals (Shao et al., 2008). However, it offers a cost effective model for preliminary hypothesis testing in the instances where analyses can be restricted to a limited number of chromosomes (Chr).

The C57BL/6J-Chr 10^A/J^/NaJ (B6.A10) consomic strain carrying A/J Chr 10 on the B6 strain background provides a useful model for studying the genetic mechanisms contributing to impaired endurance capacity of the A/J strain. A gene encoding the key enzyme of the TCA cycle, citrate synthase (*Cs*), resides on mouse Chr 10. Both human (Vollaard et al., 2009) and rodent (Holloszy, 1967) studies demonstrated that endurance training is accompanied by an increase in CS activity. Such adaptive response implies that *Cs* may play a limiting role in endurance capacity. Furthermore, A/J mice carry a strain-specific allele of the gene. We have reported that enzymatic activity of *CS* in skeletal muscle samples from the A/J strain is ~50% lower compared to B6 allele (Ratkevicius et al., 2010). As an initial step to investigating the role of the genes residing on Chr 10 in endurance capacity of the A/J mice, we used the B6.A10 consomic strain to explore relevant phenotypes.

The aim of this study was to test the hypothesis that running endurance and skeletal muscle resistance to fatigue may be reduced in the B6.A10 strain.

## Materials and methods

### Animals

All animal procedures were approved by the Lithuanian State Food and Veterinary Service (Ref. # 0230). Breeding nuclei of the C57BL/6J (B6) and C57BL/6J-Chr 10^A/J^/NaJ (B6.A10) strains were purchased from Charles River and the Jackson Laboratory, respectively. Samples of the experimental animals were bred locally.

Mice were housed in standard cages, one to three same sex animals per cage, ambient temperature 20–21°C and 40–60 % humidity, with 12-h light/ 12-h dark cycle. Animals were fed standard chow diet (LabDiet 5001) and received tap water *ad libitum*. The forced running experimental procedure on 16 B6 (8 females and 8 males) and 21 B6.A10 (7 and 14, respectively) was initiated at 12–13 week of age. Voluntary wheel running was measured in separate group of 10 B6 and 10 B6.A10 males at 15–16 week of age. The *ex vivo* testing was carried out on a separate group of 6 B6 and 6 B6.A10 males of 18–19 week of age.

### Forced treadmill running experiment

Animals were tested on a 5-lane treadmill (LE8710MAP, Panlab, Harvard Apparatus, SL, Spain) with an exercise area of 37 × 5 × 5 cm (L × W × H) in each lane, and with a built-in air puff stimulation at the end of the lane. All treadmill sessions were carried out during the dark cycle under red light illumination. Each exposure to the treadmill began with a 2-min habituation period on a stationary treadmill belt at a zero incline. Two habituation sessions of 15 min were carried out on 2 consecutive days prior to endurance test. During those sessions mice ran on the treadmill at a speed of 16.8 m/min at zero incline. The treadmill belt was cleaned with 70% ethanol between the groups.

Two days after completion of habituation mice were subjected to an endurance test. The test is a modification of the protocol described earlier (Knab et al., 2009; Massett et al., 2009). The starting speed of the treadmill at a zero incline was set at 16.8 m/min. The speed was increased by 3 m/min every 2 min until 40.8 m/min speed was reached. If a 2 min stage of 40.8 m/min speed was successfully accomplished, the speed of 40.8 m/min was maintained until exhaustion. The endurance capacity was characterized by the distance ran over the test.

Animals were motivated to run by air puffs. It was activated when the mouse touched the grid at the end of lane. If the mouse still refused to run then it was encouraged to continue running by gentle hand prodding of the tail or hindquarters. The mice readily responded to this stimulus as has been described by Steiner et al. (2011). The point of exhaustion was defined as the time (in min) at which the mouse could no longer keep pace with the treadmill despite of delivered air puffs and continuous (lasting up to 10 s) hand prodding.

### Voluntary wheel running

Animals were single housed in a cage equipped with a running wheel of 71.6 cm in inner circumference (Tecniplast, Italy). Following 1 day habituation period running wheel activity was recorded as revolutions of the wheel over 3 consecutive days. This permitted calculation of the distance ran over that period of time. However, it was not possible to determine the actual time spent on the wheel.

### Contractile properties of fast- and slow-twitch muscles

Contractility of extensor digitorum longus (EDL) and soleus muscles was examined. All procedures were carried out at room temperature (23–25°C). Following sacrifice, soleus and EDL muscles of randomly selected hind limb were excised for contractile measurements. Sutures were attached to the proximal and distal tendons of isolated muscles. Thereafter muscle was mounted between two platinum plate electrodes in 100 mL tissue bath (Radnoti, USA) filled with Tyrode's solution (in mM: 121 NaCl, 5 KCl, 0.5 MgCl_2_, 1.8 CaCl_2_, 0.4 NaH_2_PO_4_, 0.1 NaEDTA, 24 NaHCO_3_, 5.5 glucose, pH 7.4, and bubbled with 95% O_2_: 5% CO_2_). The distal tendon was attached to a stable hook and the proximal end was tied directly to the lever of the muscle test system (1200A-LR Muscle Test System, Aurora Scientific Inc., Canada). Both soleus and EDL of the same hind limb were processed in an alternating order between the animals. While processing the first muscle the other was kept in a bath with Tyrode solution. Mounted muscle was left to equilibrate in the solution for 10 min. Afterwards the muscle was stimulated using a custom-made stimulator by 25 V square pulses. It has been determined in a pilot experiment that this voltage elicits maximal contraction. Muscle length was increased and twitch force measured every 30 s until no further increase in force was observed. Muscle was kept at this optimal length (L_0_) during the subsequent experiments. Muscles were photographed with a scale in the background in order to assess muscle length with a precision of 0.1 mm. Twitch contraction time (CT) was assessed as the time elapsed from the beginning of the contraction to its peak. Twitch half relaxation time (HRT) was measured as the time taken for the force to decline to 50% of the peak value. At L_0_ muscle was subjected to 300 ms (EDL) or 900 ms (soleus) trains of pulses of increasing frequencies (20, 50, 80, 100, 150, 200 Hz). Maximal isometric force (P_0_) was estimated from plateau in force-frequency curve and usually occurred at 100 Hz for SOL and at 100–150 Hz for EDL.

Following the force-frequency procedure muscle was subjected to a fatigue protocol consisting of 180 repeated isometric contractions at 40 Hz. A train of stimuli (250 ms in duration for EDL, or 500 ms for SOL) were delivered every second for EDL and every 1.1 s for SOL, respectively. Thus, duration of fatigue protocol for EDL and SOL was 180 and 198 s, respectively. The fatigue index was calculated as the ratio between the force of the final and the first contraction of the fatigue protocol multiplied by 100.

After the measurements, the muscle was cleaned from all visible tendons, blotted, and weighed on an analytical balance (Kern, ABT 320-4M, Germany). Muscle physiological cross-sectional area (CSA) was calculated by dividing wet muscle mass by the optimal fiber length (L_f_) and the density of mammalian skeletal muscle: CSA (mm^2^) = mass (mg)/length (mm)/1.06 (mg/mm^3^) (Brooks and Faulkner, 1988). L_f_ was calculated from the L_f_/L_0_ ratio of 0.70 or 0.45 for soleus and EDL, respectively (Brooks and Faulkner, 1988). The specific tension (sP_0_) was determined by the ratio between P_0_ and CSA.

### CS enzyme activity

CS activity was measured as previously described (Ratkevicius et al., 2010). The gastrocnemius muscle samples from the B6 (*n* = 9) and B6.A10 (*n* = 9) males were homogenized in ice-cold lysis buffer (50 mM Tris·HCl, 1 mM EDTA, 1 mM EGTA, 1% Triton X-100, pH was adjusted to 7.0) with an ULTRA-TURRAX homogenizer (Rose Scientific, Edmonton, Canada). Following shaking for 60 min the homogenates were centrifuged at 13,000 g at 4°C for 10 min and the protein concentration was measured in the supernatant using the Bradford assay (Bio-Rad, Hertfordshire, UK). The reaction reagent consisted of 100 mM triethanolamine-HCl, DTNB (100 μM), 0.25% Triton-X (vol/vol), 0.5 mM oxaloacetate, 0.31 mM acetyl CoA with pH adjusted to 8.0. Ten microliters of muscle homogenate was added to start the reaction in 1000 μL. The molar extinction coefficient of 13,600 M^−1^·cm^−1^ was used to assess the maximum CS activity (Vmax) at 412 nm during the first 2 min of the reaction. The assay was carried out at room temperature (~21°C), and CS from porcine heart was used as a standard (C3260-200UN, Sigma-Aldrich, UK) for assay calibration.

### Statistical analyses

A 2-way ANOVA (Strain and Sex), 2-way ANCOVA (body weight as covariate) and Mann-Whitney *U*-test were performed where appropriate using IBM SPSS Statistics (v21) software. Mean and *SD* is presented unless stated otherwise.

## Results

### Endurance capacity

First we aimed at determining if consomic strain carrying Chr 10 of A/J strain would show a reduced performance in the forced running endurance test.

**Figure 1:**
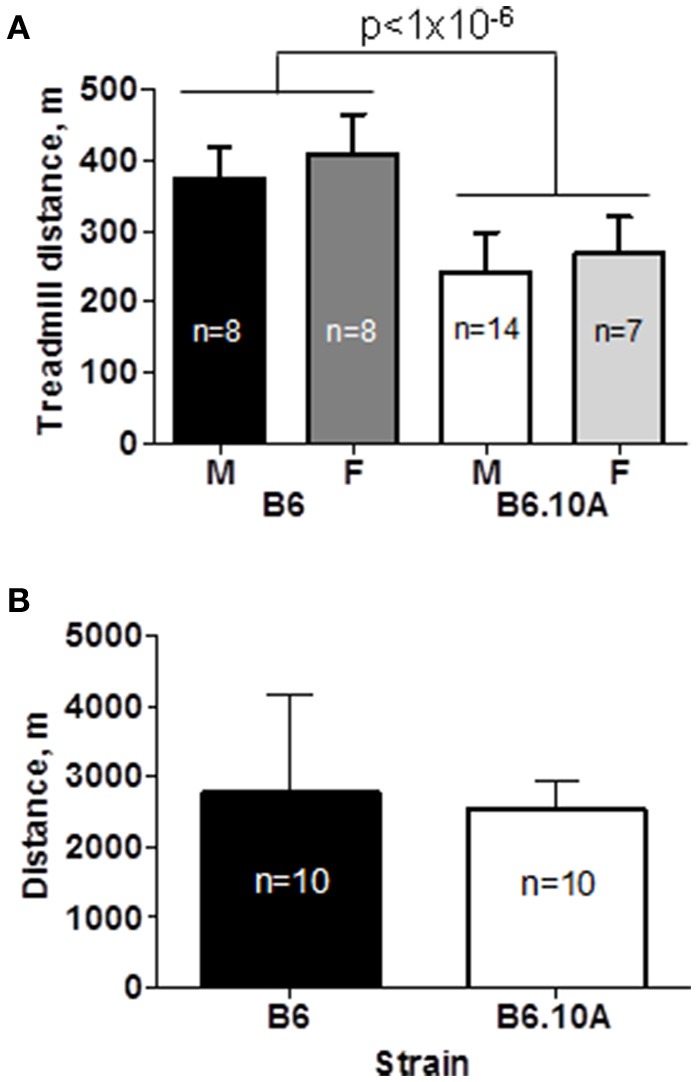
**Chromosome 10 of the A/J strain confers lower endurance capacity. (A)** Forced treadmill running distance of the consomic B6.A10 strain was reduced compared to the host C57BL/6 (B6) strain. Males (M) and females (F) were similarly affected. **(B)** Voluntary wheel running distance over 24 h period was not different between males of the two strains. Mean and *SD* shown; *n* indicates number of animals per group; *p*-value for weight adjusted strain effect is shown.

The B6 strain showed a superior endurance capacity by running significantly further compared to the B6.A10 strain, 391 ± 53 vs. 251 ± 54 m respectively (*p* < 0.0001). There was no sex effect (*p* = 0.6) on endurance performance (Figure 1A). Although the B6.A10 mice were heavier (*p* < 0.02) compared to the B6; 27.2 ± 1.9 vs. 23.8 ± 2.7 and 23.4 ±1.9 vs. 22.9 ± 2.3 g, for males and females, respectively, the strain effect on forced running distance remained statistically significant (*p* < 0.0001) after inclusion of body weight as covariate.

### Voluntary wheel running

We then wanted to examine if the difference in forced running endurance would be paralleled by a strain effect on voluntary activity.

Voluntary running capacity was assessed in male mice over the course of 72 h. Only males were studied because no sex effect was noted in the forced running test. The tested animals ran 2649 ± 996 m per day. There was no statistically significant difference between the B6 and B6.A10 strains (*p* = 0.6; Figure 1B).

### Contractile properties of isolated muscles

To test the hypothesis that peripheral mechanisms of fatigue may be contributing to poor performance in the forced running trial we analyzed contractility of isolated fast- and slow-twitch muscle of the B6 and B6.A10 males. Only males were studied because no sex effect was noted in the forced running.

Gastrocnemius weight as well as EDL weight, length, and CSA were similar between the strains, whereas soleus weight and the CSA were slightly, yet statistically significantly, larger (*p* < 0.05) in B6.A10 compared to the B6 strain (Table 1).

**Table 1:** **Properties of skeletal muscle samples of B6 and B6.A10 strain males. Mean ± ***SD*****.

	**B6 (*n* = 6)**	**B6.A10 (*n* = 6)**	***p***-value^*^
BW (g)	28.7±2.0	29.1±1.0	0.699
Gastrocnemius weight (mg)	129.8±7.8	138.8±5.4	0.065
Soleus weight (mg)	8.6±0.9	9.7±0.4	**0.015**
Soleus L_0_ (mm)	13.9±0.6	14.1±0.5	0.485
Soleus L_f_ (mm)	9.9±0.4	10.0±0.4	0.589
Soleus CSA (mm^2^)	0.9±0.1	1.0±0.1	**0.041**
EDL (mg)	10.5±1.2	11.1±0.6	0.394
EDL L_0_ (mm)	15.9±0.5	15.9±0.7	0.699
EDL L_f_ (mm)	7.2±0.2	7.2±0.3	0.699
EDL CSA (mm^2^)	1.6±0.2	1.6±0.0	0.818

* *Mann-Whitney U-test between the strains, exact significance displayed; values <0.05 are highlighted in bold. BW, body weight; L_0_, optimal length; L_f_, optimal fiber length; CSA, physiological cross-sectional area; n indicates number of samples tested*.

**Figure 2:**
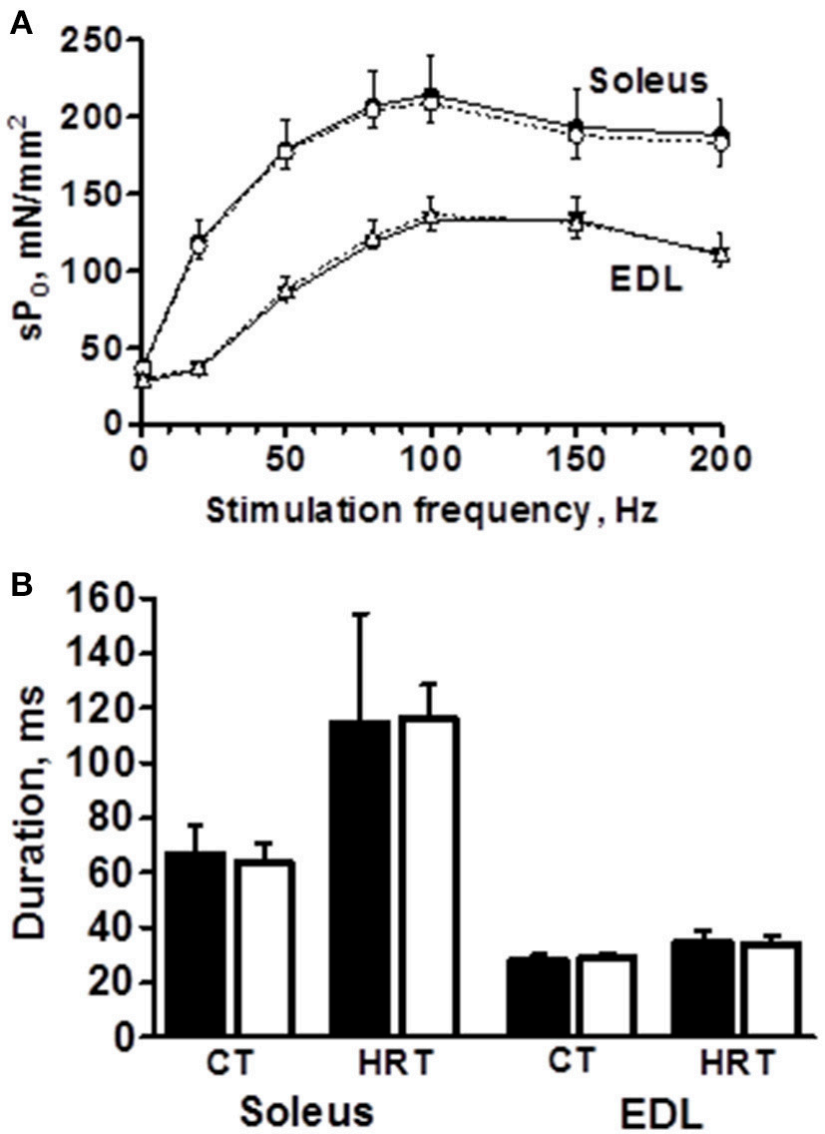
**Contractile properties of isolated slow- (soleus) and fast-twitch (extensor digitorum longus, EDL) muscles are not associated with chromosome 10 genotype**. Force-frequency relationship expressed as specific force **(A)**, contraction (CT) and half relaxation times (HRT) of single twitch **(B)** are shown. Black symbols/bars represent B6 and white B6.A10 strain. Mean and *SD* shown.

**Figure 3:**
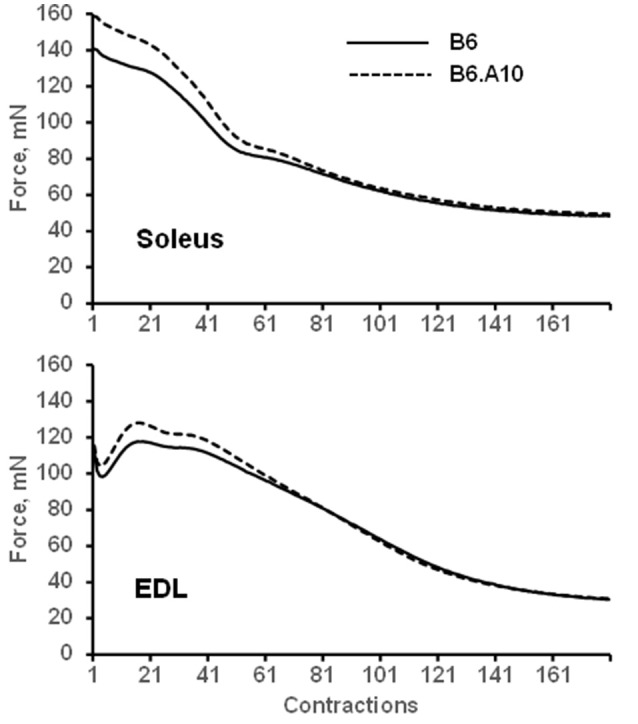
**Fatigue of isolated muscle is not linked to chromosome 10 genotype**. Mean force of 180 repeated contractions of slow-twitch soleus **(top)** and fast-twitch EDL **(bottom)** muscles are shown. Solid and dashed lines represent B6 and B6.A10 strains, respectively.

The peak force of isometric contraction was similar between the B6 and B6.A10 strains in both soleus, 192 ± 24 vs. 211 ± 10 mN (*p* = 0.13), respectively, and EDL muscles, 209 ± 21 vs. 217 ± 13 mN (*p* = 0.59). The isometric contraction force after adjustment for the CSA of the muscle did not differ between the strains in either soleus or EDL muscle (Figure 2A). The temporal properties of twitch contraction and relaxation did not differ between the strains either (Figure 2B). A series of repeated contractions in both fast- and slow-twitch muscle resulted in a similar level of fatigue between the strains (Figure 3). Fatigue index in B6 and B6.A10 soleus was 34 ± 5 and 31 ± 3%, respectively (*p* = 0.24), and 27 ± 2 and 27 ± 4% (*p* = 0.7) in the EDL.

### CS enzymatic activity

The CS activity in gastrocnemius muscle was assessed to examine if it could have contributed to reduced forced running endurance of the B6.A10 strain. The analysis indicated that CS activity was significantly lower (*p* < 0.0001) in B6.A10 compared to B6 strain, 487.7 ± 69.5 vs. 754.0 ± 68.6 U/g, respectively.

## Discussion

The main findings of this study are that Chr 10 of the A/J strain is associated with substantially lower endurance capacity compared to the B6 strain. The between strain difference remained robust and highly significant even following adjustments for body weight. Consistently with our recent observation in a separate study involving the same strains (Kilikevicius et al., 2016), it also emerged that soleus muscle weight was higher in B6.A10 compared to the B6 mice.

Endurance capacity of the B6 strain measured in the present study as the distance run during the test was comparable to values in the literature despite the differences in the testing protocol and age (Massett, 2013; Courtney and Massett, 2014). In these earlier studies endurance capacity of the A/J strain equaled to just ~43% of that in the B6 strain. Our model, showing that B6.A10 mice ran only 64% of the distance completed by B6, implicates Chr 10 gene(s) in impaired endurance of the A/J strain. This novel finding adds to the previous knowledge of the role of Chr 14; specifically, males of consomic strain B6.A14 ran ~84% of the distance completed by B6 strain (Courtney and Massett, 2014). Thus, it emerges that the difference in endurance between the B6 and A/J strains is polygenic in nature, caused by chromosome 10 and 14 genes; the identity of the causative genes, however, remains to be determined.

Physical activity is an important determinant of health and fitness. Voluntary wheel running is extensively used for quantification of physical activity in rodents. The majority of rodent species avidly engage in this locomotor activity although it is debatable what motivates animals to run (Careau et al., 2012). Although both voluntary and forced running represent locomotor activities, mechanisms limiting performance under these two conditions are likely to differ. The forced running test is continuous and limited by the neuromuscular and/or cardiovascular systems, while the voluntary running consists of bursts of activity accumulated over a long period of time and hence is not determined by the same physiological mechanisms. Such a notion is consistent with the observations that voluntary running distance did not differ markedly between the B6 and A/J strains (Lightfoot et al., 2010; Courtney and Massett, 2012). A similar uncoupling of performance in the two types of activity was also observed between other strains; for instance 129S1/SvImJ mice performed poorly in voluntary running compared to the B6 but matched their performance in forced running (Lightfoot et al., 2010; Courtney and Massett, 2012). Thus, mechanisms limiting forced running endurance in the B6.A10 strain do not affect voluntary running of these animals.

Enzymatic activity of citrate synthase (CS) has been used as a biomarker of mitochondrial content (Larsen et al., 2012) and function (Jacobs et al., 2013) in skeletal muscle. It has been known that endurance exercise training leads to an increase in CS activity in exercised skeletal muscles (Holloszy and Booth, 1976). The CS encoding gene, *Cs*, resides in the telomeric region of mouse Chr 10. The A/J strain is characterized by ~50% reduced enzymatic activity of CS in skeletal muscle (Ratkevicius et al., 2010). Consistently with that, albeit a smaller reduction of ~35% was observed in the present study in B6.A10 strain carrying the A/J strain Chromosome 10. This discrepancy between the A/J and B6.A10 strains suggests that ~35% reduction can be attributed to Chromosome 10, whereas the remaining difference is due to the mechanisms governed by the factors out with Chr 10, which could affect quantity of the enzyme in the muscle (e.g., by influencing proportion of oxidative fibers) rather than the innate CS activity.

We hypothesized that the impaired endurance capacity of the B6.A10 strain might be a reflection of fatigue resistance of skeletal muscles. However, fatigability of the muscles was not strain dependent. To understand this unexpected observation we first considered whether sample size could have been too small. That, however, is unlikely. If the strain effect on the fatigue index of isolated muscle was proportional to that on the forced running endurance, we had statistical power >85% for detecting it at alpha level of 1% in this sample. The second possible explanation is methodological. It has been demonstrated that isolated muscles fatigue faster compared to individual muscle fibers dissected from the same muscle because of the restricted oxygen diffusion to the core of the muscle (Zhang et al., 2006). If differences in the aerobic metabolism of skeletal muscle were responsible for accelerated fatigue in the forced running test (a plausible hypothesis considering the difference in CS activity between the B6 and B6.A10), its relevance might have been substantially diminished under the hypoxic conditions where contractility would shift toward reliance on anaerobic metabolism. Examining fatigue properties in isolated muscle fibers and/or the whole muscle *in situ* would be required to address this question. The third alternative explanation is that forced running endurance could be primarily limited by cardiovascular function. The echocardiographic variables (ventricular wall thickness, chamber size) in the B6 strain exhibit properties reminiscent of the “athlete's heart” in comparison to A/J (Hoit et al., 2002). The estimates of the cardiac output based on the systolic-diastolic volume difference and heart rate are ~15% lower in the A/J strain compared to the B6 (Hoit et al., 2002; Lake et al., 2009; The Jackson Laboratory, 2010). However, as ventricular parameters of the B6.A10 strain are not known, its direct comparison to the B6 strain is not possible. Further studies will be required to prioritize between the contribution of cardiac and muscular mechanisms to the difference in fatigability between the B6 and B6.A10 mice.

A difference in soleus muscle weight between the B6 and B6.A10 strains (Table 1) provides further support to recently reported observation between the same strains (Kilikevicius et al., 2016). Albeit a ~13% increase in soleus of B6.A10 strain is less extensive than ~40% reported in that study, collectively these findings are consistent with a notion that A/J variant of one or more chromosome 10 genes confer an increase in soleus weight. The direction of the effect, i.e., B6.A10 > B6, is somewhat surprising because in the B6 vs. A/J comparison the former exhibits higher weight due to more numerous fibers (Kilikevicius et al., 2013). The contractile function measured in the present study was comparable with literature, both for the soleus (Brooks and Faulkner, 1988) and EDL (Amthor et al., 2007) muscles. In general, there was no difference between the B6 and B6.A10 strains in contraction and relaxation times (Figure 2), although peak isometric force in B6.A10 soleus was slightly elevated mirroring muscle weight difference between the strains. The mechanisms and gene(s) underlying the strain difference remain to be determined.

In conclusion, Chr 10 of the A/J strain contributes to the reduced CS enzymatic activity and endurance performance. The effect may be mediated by the cardiovascular function, although the role of skeletal muscle cannot be ruled out. An intercross between the B6 and B6.10A strains provides an attractive research model for further analyses and identification of gene(s) determining endurance capacity.

## Author contributions

AL, AR, and TV designed the study; MK carried out forced running and voluntary wheel running experiments with supervision of TV; PM performed physiological analyses of isolated muscles; AF measured enzyme activity; AL and MK wrote the manuscript with contribution of all authors.

### Conflict of interest statement

The authors declare that the research was conducted in the absence of any commercial or financial relationships that could be construed as a potential conflict of interest.
